# Intensive measures of luminescence in GaN/InGaN heterostructures

**DOI:** 10.1371/journal.pone.0222928

**Published:** 2019-09-24

**Authors:** Jui-Ju Hsiao, Yi-Jen Huang, Hung-Ing Chen, Joe-Air Jiang, Jen-Cheng Wang, Ya-Fen Wu, Tzer-En Nee

**Affiliations:** 1 Graduate Institute of Electro-optical Engineering and Department of Electronic Engineering, Chang Gung University, Kwei-Shan, Tao-Yuan, Taiwan, Republic of China; 2 Department of Biomechatronics Engineering, National Taiwan University, Taipei, Taiwan, Republic of China; 3 Department of Electronic Engineering, Ming Chi University of Technology, Taishan Dist., New Taipei City, Taiwan, Republic of China; 4 Department of Oral and Maxillofacial Surgery, Linkou Chang Gung Memorial Hospital, Kwei-Shan, Tao-Yuan, Taiwan, Republic of China; Indian Institute of Science Education and Research (IISER), INDIA

## Abstract

The intensive measures of luminescence in a GaN/InGaN multiple quantum well system are used to examine the thermodynamics and phenomenological structure. The radiative /nonradiative transitions along with absorbed or emitted phonons that occur between the different quantum states of the electrons and holes associated with these processes make the quantum efficiency of a semiconductor nanosystem in an equilibrium state an extensive property. It has long been recognized that tuning of the indium (In) composition in InGaN interlayers gives the potential to obtain a spectrum in the near-infrared to near-ultraviolet spectral range. The thermodynamic intensive properties, including the Debye temperature, carrier temperature, and junction temperature, are the most appropriate metrics to describe the optical-related interactions inherent in a given heterostructure and so can be used as the state variables for understanding the quantum exchange behaviors. The energetic features of the quantum processes are characterized based on analysis of the intensive parameters as determined by means of electroluminescence (EL) and photoluminescence (PL) spectroscopy and current-voltage measurement and then correlated with the designed InGaN/GaN microstructures. According to the McCumber-Sturge theory, the EL and PL Debye temperatures obtained experimentally signal the strength of the electron-phonon and photon-phonon interaction, respectively, while the EL and PL carrier/junction temperatures correspond to the carrier localization. Higher EL Debye temperatures and lower EL carrier/junction temperatures reflect significantly higher luminescence quantum yields, indicative of electron-phonon coupling in the transfer of thermal energy between the confined electrons and the enhancement by excited phonons of heat-assisted emissions. On the other hand, the observation of low luminescence efficiency, corresponding to the lower PL Debye temperatures and higher PL carrier/junction temperatures, is attributed to photon-phonon coupling. These findings are in good accordance to the dependence of the EL and PL quantum efficiency on the In-content of the InGaN/GaN barriers, suggesting that the characteristic Debye and carrier/junction temperatures are intensive parameters useful for assessing the optical properties of a nano-engineered semiconductor heterostructure.

## Introduction

Advances in semiconductor nanoscience and nanotechnology will allow for continued rapid progress towards the development of an improved understanding of the fundamental physical principles governing the quantum processes at the nanoscale level [1–3]. Both reduced dimensionality and device scaling lead to increased problems of heat management in heterostructures. As a result, the phonon-related interactions associated with electrons and photons become important design issues for a wide range of technologies, such as for microwave devices, high-k devices, metal-oxide-semiconductor field-effect transistors (MOSFETs) with high-k gate oxides, solar cells, light-emitting diodes, and laser diodes [4–8]. Indeed, significant changes in the dispersion curves of the elementary excitations which are reflective of modulation of the phonon states produced by the downscaling size-reduction technology enable the tailoring of the characteristics of the heteromaterial to a previously unprecedented extent. Over the past decade, combining computer science with quantum mechanics has led to rapid development of quantum computing. Since electrons not only couple to photons but also interact with phonons, consideration of phenomenon such as energy dissipation, decoherence and carrier thermalization have become critical for realistic quantum nanodevice design in thermal phonon bath engineering and quantum reservoir engineering in terms of solid state quantum information processing [9, 10]. Furthermore, as far as the charge transport behaviors, including the electrical and thermal conductivity and the thermal related electricity in nanoscale condensed structures are concerned, it was well known that, in addition to the diffusion and external fields, scattering theory inherently governs the effects of the dissipation processes, that is the transfer of energy from the electron system to the phonon system [11]. Dielectric engineering and mobility engineering are both attractive routes for improving the performance of nanostructured photonic and electronic heterodevices [12].

Although the fundamentals of light-matter interactions in solid state systems have been well-studied, very little research has been done to explore the interrelationships between the intensive parameters obtained and the experimental results observed in nanometric space in response to electrical or optical excitations [13, 14]. It is expected that using the intensive measures to phenomenologically characterize the optical processes in organic or inorganic-based structures will facilitate the development of more efficient optoelectronic devices. Indeed, intensive research of nanosystems is becoming increasingly appealing not only because it will establish the connection between incongruous results often reported by different groups for seemingly identical materials, but also because it will allow a plethora of applications in modern functional systems such as microelectronic, magnetic, mechanical, thermal and even biological systems [15, 16]. In this work, we employ GaN/InGaN multiple quantum well heterostructures to experimentally unveil, from the temperature-dependent current-voltage (IV) characteristics, photoluminescence (PL), and electroluminescence (EL) for a wide temperature range from 20 to 300 K, the different thermobehaviors as a function of the In content in barriers. In order to better understand the thermal localizations of carrier distributions, it is necessary to extract the emission-related carrier and junction temperatures for both the EL and PL from the high-energy band tails and wavelength shifts of the InGaN luminescence spectra, respectively, while the IV-junction temperature is obtained from the forward bias of the IV measurements [17–19]. Furthermore, the injection of electrons and impingement of photons associated with the anharmonic phonon potential of the heterocrystalline structure give rise to the electron-phonon and photon-phonon interactions, respectively, so they can feasibly be treated as perturbation of the harmonic phonon states [20]. However, knowledge of the coupling strength between the energized particles and the anharmonic phonons is scanty, and, in the present work, might be obtained from intensive quantity-related arguments about the macroscopic phenomena. With the help of the Debye model, the analyses of the thermal variations of EL and PL linewidths and wavelengths enable us to extract the EL and PL Debye temperatures, respectively, while the McCumber-Sturge theory is used to describe the electron-phonon and photon-phonon interactions in conjunction with the luminescence experiments. To corroborate the robustness of our proposed scheme, we also characterize the dependence of the quantum efficiency of EL and PL on the In molar fraction in the InGaN/GaN interlayers.

## Experiments

**Fig 1:**
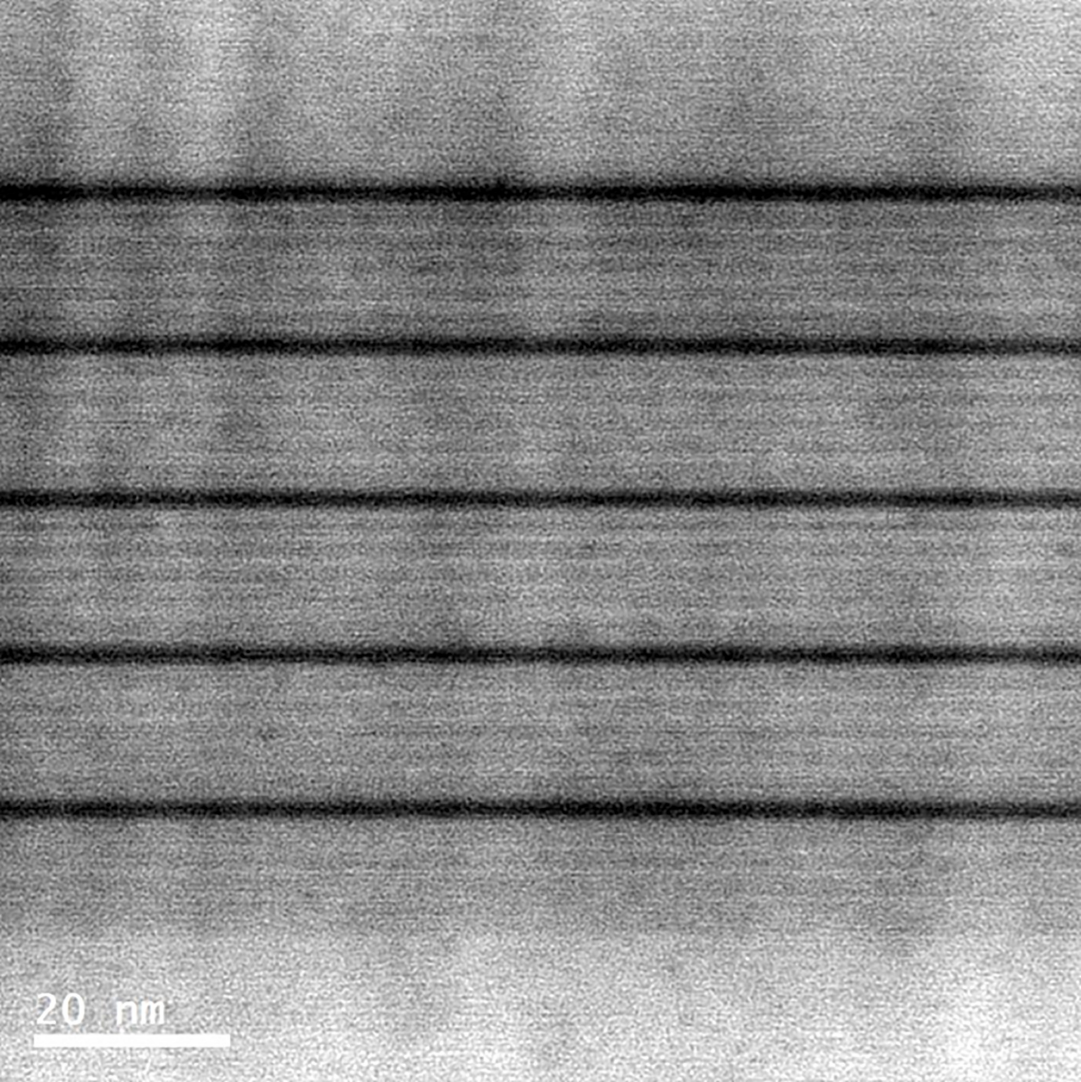
The cross-sectional TEM image for GaN/InGaN multiple quantum well heterostructures. In the MQWs heterostructure, the thicknesses of the InGaN well and GaN barrier were chosen to be 2 nm and 11 nm for the heterostructure samples.

The GaN/InGaN multiple quantum well heterostructures used in this work were grown on c-plane sapphire substrates using a metal organic vapor phase epitaxy (MOVPE) system. Fig 1 shows the cross-sectional TEM image for GaN/InGaN multiple quantum well heterostructures, with a 20-nm-thick GaN buffer layer, a 3-μm-thick n-type GaN:Si, followed by an undoped GaN layer with five periods of In_0.15_Ga_0.85_N multiple quantum wells (MQWs) heterostructure, and a 150-nm-thick p-type GaN:Mg cap layer. In the MQWs heterostructure, the thicknesses of the InGaN well and GaN barrier were chosen to be 2 nm and 11 nm for the heterostructure samples. We prepared three kinds of MQWs heterostructures, with different types of five-period InxGa1-xN/GaN multiple quantum barriers (MQBs) in the active regions of these devices. It structure identical to that of all the samples, except the MQBs are replace by indium increasing composition-graded InxGa1-xN MQBs with the linearly graded indium composition x = 0.005, 0.01, and 0.02, respectively. The X-ray diffraction (XRD) line profile analyses are as the same as our previous reports, not shown here [21]. The Debye model eventually allows for a quantitative description of the thermodynamics of a crystal. It is well known that the Debye temperature can be used to represent the crystal structures and the consequent properties of the elementary interactions in the real matters [22].

The temperature-dependent forward voltages were extracted from current-voltage measurements. A Keithley 2423 was a used as current source to drive the samples mounted in a closed-cycle helium cryostat over a broad temperature range, from 300 to 20 K. The temperature-dependent electroluminescence (EL) characteristics of the experimental heterostrucutres were obtained for samples mounted in a closed-cycle He cryostat with an excitation current of 20 mA over a broad temperature range from 300 to 20 K. For the photoluminescence (PL) measurements, the heterostrucutres were mounted in a closed-cycle He cryostat. The PL measurements were taken every 20K over temperature range from 300 to 20 K. The samples were excited by a cw He-Cd laser (325 nm) prior to the PL experiments. The luminescence signal was dispersed by an Acton SpectraPro 500i monochromator for detection by a Si photodiode employing the standard lock-in amplification technique.

In a previous report Xi *et al*. described the relation between the forward voltage and the junction temperature [23]. The calibration measurement serves as a reference, the junction temperature under different DC current can be found by

$$T_{j}=\frac{\left(V_{f}-A\right)}{B},$$ (1)

where *T*_*j*_ is the junction temperature; *V*_*f*_ is the forward voltage and A and B are the fitting parameters. The junction temperature can also be described by the peak shift. On the other hand, the high-energy variation follows the proportionality

$$I\propto \exp\;\left(-\frac{hv}{kT_{c}}\right),$$ (2)

where *T*_*c*_ is the carrier temperature; *hv* is the photon energy and *k* is the Boltzmann’s constant. Based on the zero-phonon model, the McCumber-Sturge theory can be used to deduce the photon-phonon and electron-phonon interaction in heterosystem with the spectroscopic experiments [24]. The change both in peak shift and in the FWHM with temperature can be expressed in similar form as

$$\Delta \xi \left(T\right)=\alpha {\left(\frac{T}{\theta_{D}}\right)}^{a}\int_{0}^{\frac{\theta_{D}}{T}}\frac{x^{a-1}e^{x}}{{\left(e^{x}-1\right)}^{2}}dx,$$ (3)

where Δ*ξ*(*T*) is the temperature-dependent spectral observations; *α* is the phonon interaction constant with electron or photon. Here, *θ*_*D*_ is the Debye temperature, *T* is the measuring temperature and *a* is the power law index. The value of the power law index, i.e., *a*, is dependent on the spectral behavior of concern. The power law indices representing the peak shift and the line broadening were equal to 4 and 7, respectively. Furthermore, the electrical injection and optical excitation will cause differ valuable insight for description of thermal properties in term of Debye model as well as on the property one is modelling. The various Debye temperatures usually differ by less than 10–20%. The Debye temperatures depend on the temperature obtained by PL and EL spectra measurement may give valuable intensive parameters [25]. In summary, we offer an alternative way to directly obtain the intensive values from the current-voltage curve, PL and EL spectral measurements.

## Results and discussion

**Fig 2:**
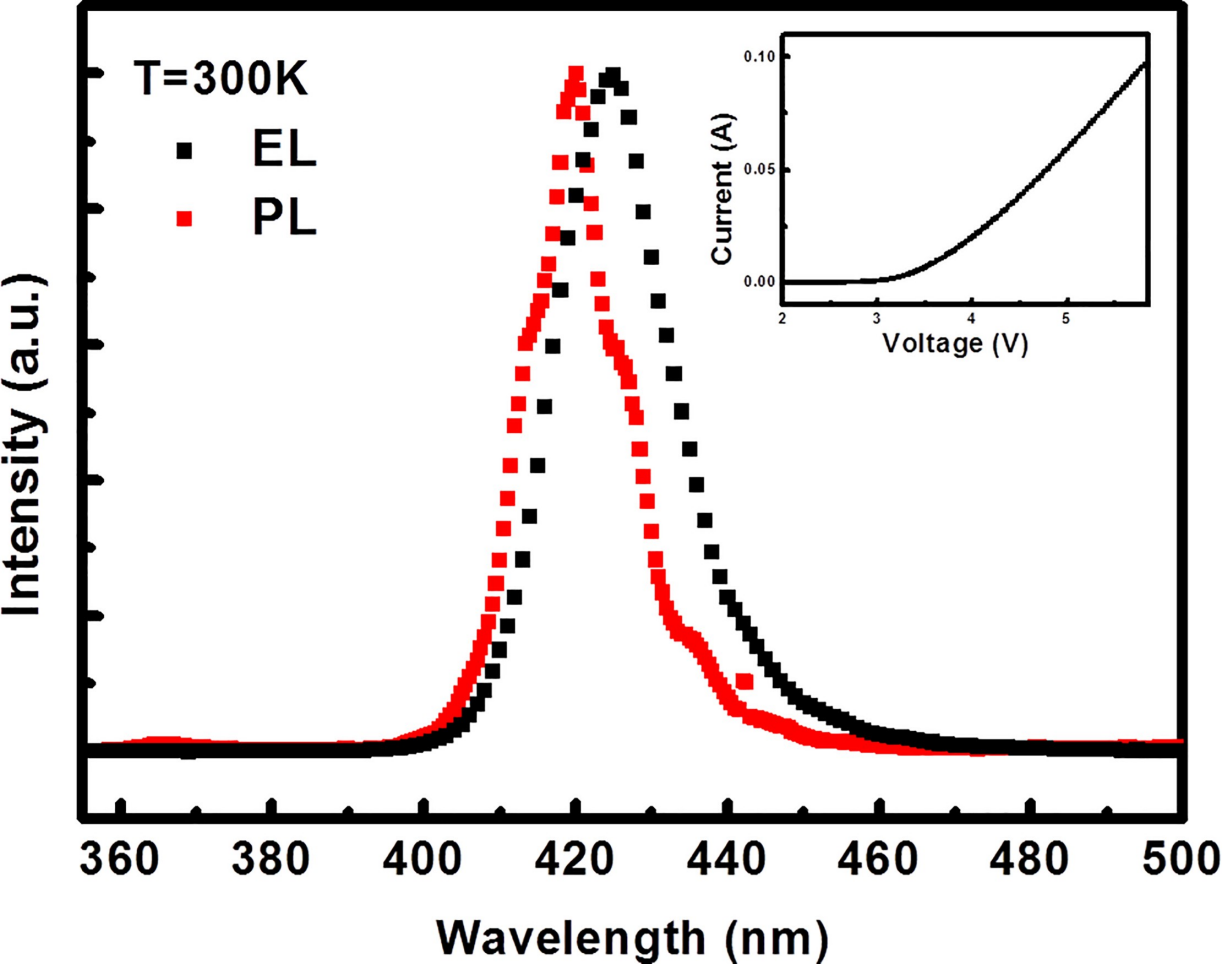
The electroluminescence (EL) and photoluminescence (PL) spectra of GaN/InGaN heterostructures at an ambient temperature of 300 K. The EL spectrum was measured under a direct injection current level of 20 mA.

Fig 2 shows the photoluminescence (PL) and electroluminescence (EL) spectra of GaN/InGaN heterostructures at an ambient temperature of 300 K. The EL spectrum was measured under direct injection current level at 20 mA. The inset shows the current-voltage curve of GaN/InGaN heterostructures at 300 K. The PL and EL spectra clearly reveal that the InGaN emission peaks located at around 424 and 432 nm, respectively. This is indicative of the difference in the quantum confinement process between photo-excited and electro-excited luminescence measurements of the GaN/InGaN heterostructure. The results can be explained by attributing the PL to an energy transfer process, while the EL emission can also be explained by direct electrons and holes trapping [26, 27]. However, the same heterostructure may also produce different experimentally signal depending upon the excitation source. The results means that the electrical and optical properties may affect quantum process in a GaN/InGaN multiple quantum well system. The quantum interaction between the electrons, photons, and phonons of GaN/InGaN heterostructures were investigated by measuring the intensive parameters under electrical injection and optical excitation. In order to deeply realize the quantum exchange behaviors in the GaN/InGaN multiple quantum well system, we further examines the thermally-related PL and EL spectroscopies and DC current-voltage characteristics.

**Fig 3:**
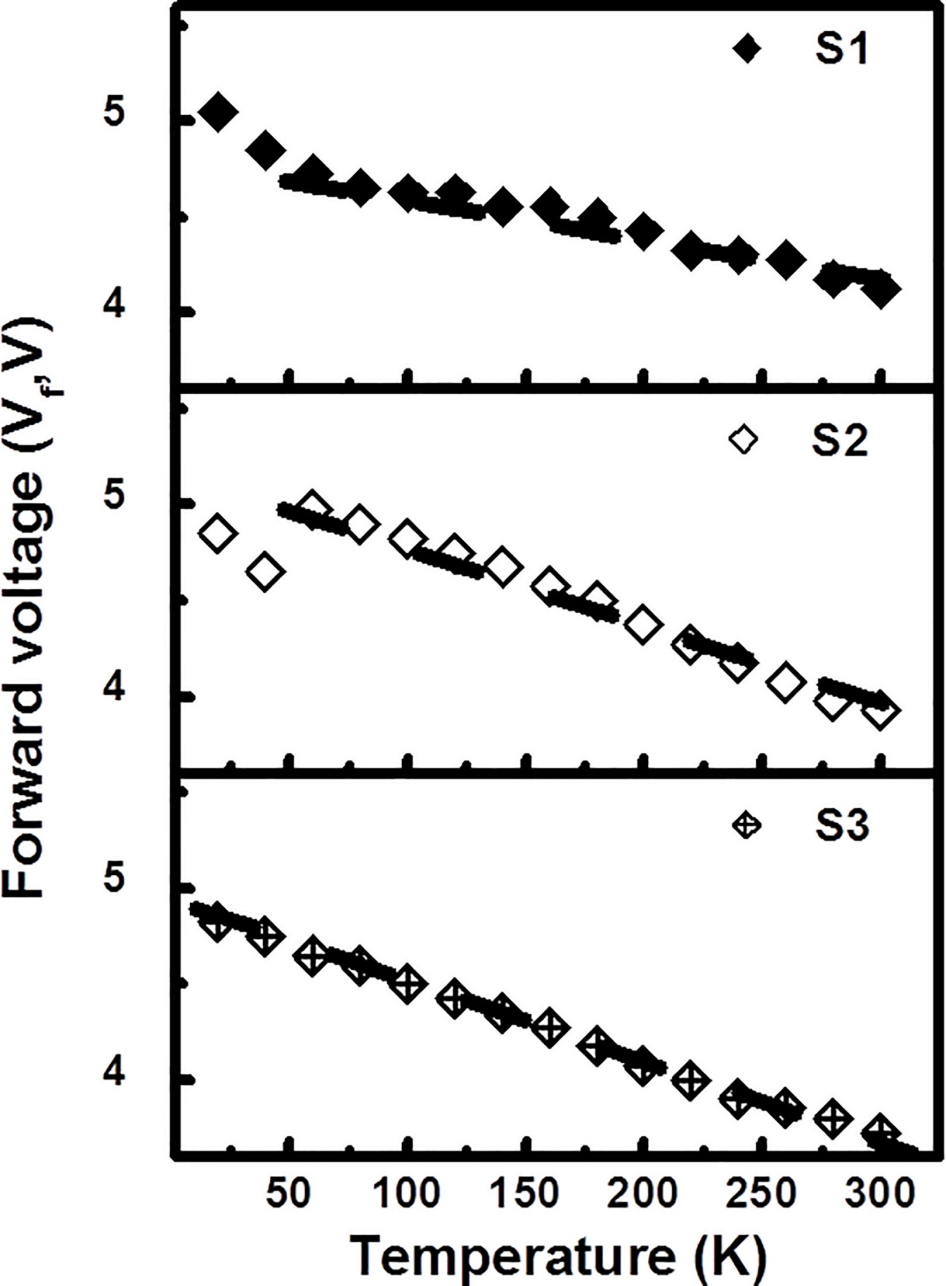
The current-voltage characteristics of GaN/InGaN heterosystems with increasing InN molar fractions (S1 to S3) measured at an injection current level of 20 mA.

The current-voltage characteristics of GaN/InGaN heterosystems with increasing InN molar fractions (S1 to S3) were measured at an injection current level of 20 mA. The variations in the forward voltage obtained in response to changes in temperature are also shown in the experimental observations summarized in Fig 3. The slope of the forward voltage (V_f_) versus temperature was estimated to be -2.26, -4.1, -4.6 mV/K, respectively, implying larger resistive contributions with increasing In incorporation in the heterostructure at elevated temperatures. An increase in the InN molar fraction of the In_x_Ga_1-x_N/GaN multiple quantum barriers (MQBs) in the active regions caused microstructure impurity-induced disordering to arise, thereby decreasing conductivity, as expected. In addition, temperature-dependent conductance also occurred as the carriers hopped through the In segregation-induced static MQBs [28, 29]. As a consequence, the slope of the forward voltage versus temperature was highest for S3 compared with the other low In-content interlayer samples at an elevated temperature. From the results we can see that the poor thermal conductivity of the epoxy generated heat retention in the device. The shift in the forward voltage in response to the changes of temperature was due to the Shockley contact related resistance in the chip and the band gap shrinkage effect [30].

**Fig 4:**
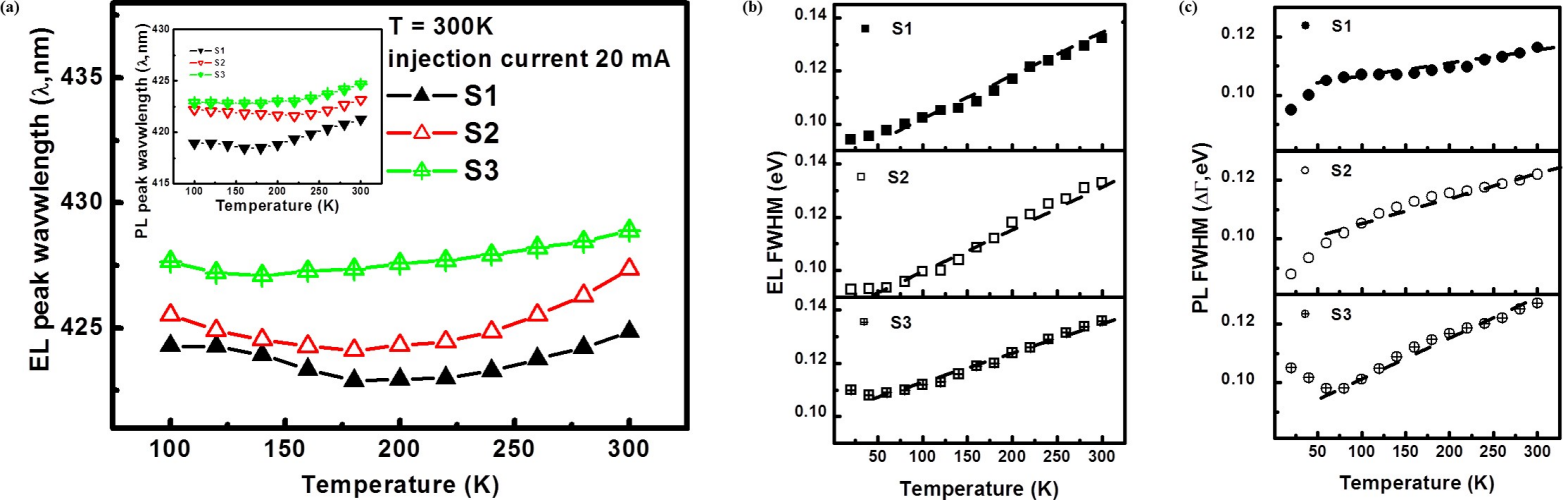
(a) The emission peak energy from EL and PL measurements for GaN/InGaN blue MQB heterostrucutres at different temperatures. (b, c) The thermal-related FWHM from EL and PL measurement experiments can be used to study the thermal effects of GaN/InGaN heterstructures, of the photon-electron interaction with the lattice.

Fig 4 shows the emission peak energy and the full width at half maximum (FWHM) for the EL and PL measurements for GaN/InGaN blue MQB heterostructures obtained at different temperatures. In each case, the excitation power for EL measurement was fixed at 20 mA. The emission peak energy and FWHM for the EL and PL spectra for the GaN/InGaN heterosystem, S1 to S3 (caused by increasing the In composition and as a function of temperature) were obtained using a Gaussian fit method. It is apparent from the plot that the emission peak energy changed inherently with respect to the InN molar fraction of the GaN/InGaN barrier structures. The thermal-related shift in the emission peak wavelength to longer wavelengths increased after 200 K, as can be seen in Fig 4(A). The experimental observations show that the emission peaks display a linear correspondence at high temperature. A shift in the peak position of the conduction and valence bands is due to the temperature-dependent dilatation of the lattice, indicating temperature-induced bandgap shrinkage of the ternary GaN/InGaN heterostructures [31]. In the low temperature region, below 200 K, the emission peak showed a nonlinear relationship as a function of temperature. Indeed for a number of diamond structure solids it even becomes negative [32]. The temperature-dependence of the EL FWHM from S1 to S3 were 0.142, 0.128, 0.106 meV/K, respectively; those same values were 0.052, 0.083, 0.104 meV/K, respectively for the PL measurement, as shown in Fig 4(B) and 4(C). From the results, we found that there was a decrease in the EL thermally-related FWHM with an increase in the InN molar fraction incorporated into the GaN/InGaN interlayers. However, there was an increase in the temperature-dependent PL FWHM from S1 to S3. In addition, the trend was for the shift in the emission peak with tuning of the indium (In) composition to be the same as the change of broadening of the experimental EL and PL observation. Moreover, we found the temperature-dependence FWHM of the EL and PL measurements of GaN/InGaN heterostructures with tuning the indium (In) composition obtained from the experiments can be used to study the thermal effect, the electron and photon thermalization process arising from interaction with the vibrating lattice.

**Fig 5:**
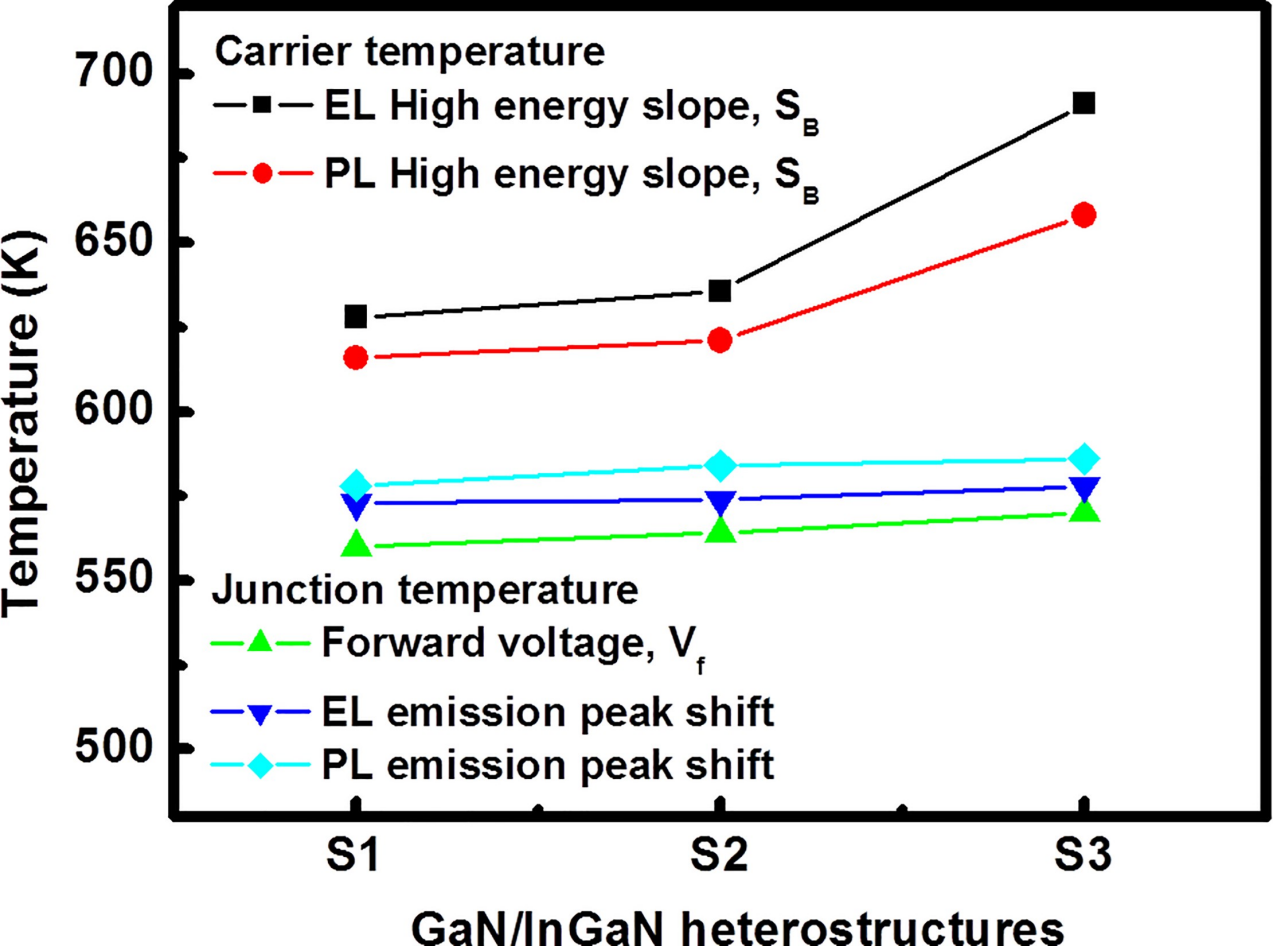
The EL and PL junction temperatures obtained from the forward voltage and wavelength shift techniques and the EL and PL carrier temperatures estimated from the high-energy band tails of the InGaN luminescence spectra for GaN/InGaN blue MQB heterostrucutres.

In order to deeper understanding of the thermodynamic intensive parameter of luminescence characteristics in GaN/InGaN heterostructures, we further compare the carrier/junction temperatures, as shown in Fig 5. The EL and PL carrier temperatures were estimated from the high-energy band tails of the InGaN luminescence spectra, with the carrier distribution approximated by using the Boltzmann distribution. The effective carrier temperatures, estimated from related EL observations for samples with increasing In composition in the InGaN interlayers, were 628, 636 and 691 K, respectively; for the PL observation they were 616, 621 and 658 K, respectively. The forward voltage and wavelength shift techniques were used to obtain the EL and PL junction temperatures. The carrier temperature measured by using high-energy band tail is higher than junction temperature. It is possible to assume that high forward voltage and high-energy injection of carriers into the active region [33]. In Fig 5, it can be seen there was an increase in the junction temperatures extracted with the forward voltage method from 560 to 570 K as the In mole increased in the InGaN heterostructures. The EL junction temperature, as deduced from the wavelength shift method, increased from 573 to 578 K with the increase in the In mole fraction in the InGaN heterostructures; and for the PL observation the values increased from 578 to 586 K. The carrier temperature determined from the high-energy band tail is more accurate than junction temperature obtained from the wavelength shift method. This is due to the spectral linewidth increase; decrease the high-energy band tail, thereby increase the carrier temperature. It is commonly accepted that the emission peak value is less of the spectral linewidth. From the experimental results, we can see that the carrier/junction temperature increased with the increase in the In mole fraction in the interlayer. This is attributed to the confinement region whose thermal localization of carrier distributions dependence with increases In mole fraction in barriers. However, the EL carrier temperatures are higher than PL carrier temperatures, indicating that the effect of an external electric field causes the poor carrier confinement in the MQW interlayer. These results indicate that the injection of electrons and impingement of photons associated with the anharmonic phonon potential of the heterocrystalline structure is different.

**Fig 6:**
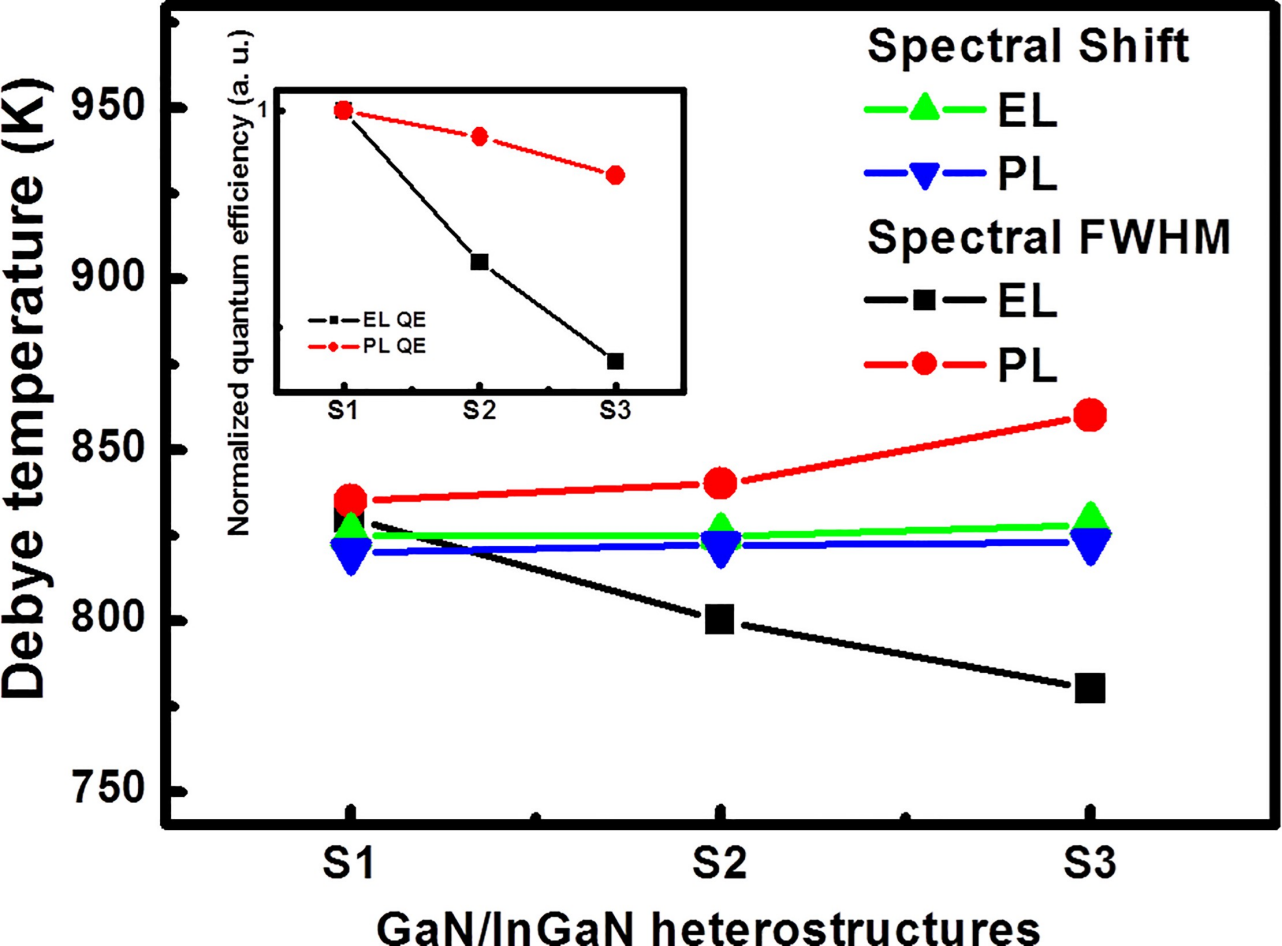
The EL and PL Debye temperature extracted from the observations of the FWHM and red-shift in the emission peak as a function of tuning of the In composition in InGaN interlayers. The inset shows, the dependence of the observed EL/PL related quantum efficiency (QE) on the variation of the indium composition for S1 to S3.

With the help of the Debye model, the analyses of the thermal variations of EL and PL linewidths and wavelengths enable us to extract the EL and PL Debye temperatures, respectively, while the McCumber-Sturge theory is used to describe the electron-phonon and photon-phonon interactions in conjunction as a function of the tuning of the In composition in InGaN interlayers, as plotted in Fig 6. The Debye temperatures, estimated from the temperature-dependent redshift-related peak observed in the EL observation were 825, 826, and 828 K, respectively; for the PL observation were 820, 822, and 823 K, respectively. Furthermore, the Debye temperatures, estimated from the temperature-dependent FWHM related EL observations for sample with increasing In composition in InGaN interlayers were 830, 800 and 780 K, respectively; for the PL observation they were 835, 840 and 860 K, respectively, as shown in Fig 6. From the results, we can found that the Debye temperature as a function of broadening variation in response to change in temperature under electrical injection and optical excitation are different with increasing In composition in InGaN/GaN interlayer samples. The redshift-related Debye temperature changed is not obvious here; we infer that the non-radiative recombination is dominant and carrier’s lifetime is almost constant after 200 K, leading to the temperature-induced bandgap shrinkage becoming responsible for the redshift in the emission peak. As can be seen in the inset to Fig 6, the observed EL/PL related quantum efficiency was dependent on the variation of the indium composition, as ours previous study [34]. It is interesting to describe a functional relationship between thermodynamic intensive parameter analysis for GaN/InGaN MQW systems subjected to electrical injection and optical excitation. Base on above results, the FWHM-related Debye temperatures reduced and the carrier/junction temperatures increased by electrical injected produce significantly drop luminescence quantum yields though variation of the In composition, indicative of electron-phonon coupling in the transfer of thermal energy between the confined electrons and the excited phonons enhancing the heat-assisted emissions. On the other hand, observed that the PL luminescence efficiency is higher than EL luminescence efficiency. It is possible to assume that PL Debye temperatures increase enhancement photon-phonon interaction in the quantum efficiency. Referring to the reports by Li *et al*. for Electroluminescence properties of InGaN/GaN multiple quantum well-based heterostrucutres with different indium contents and different well widths [35], using the experiment data with different kind of heterostructure configuration to estimated intensive quantities were shown in Table 1, the reference reports results same as our research. The results are in good agreement with the experimentally expected value ranges and demonstrate the transfer of thermal energy process on the heteromaterial properties. We demonstrated an easy way to know the luminescence characteristics as possible using intensive parameter.

**Table 1:** The values of normalized quantum efficiency, Debye temperature, junction/carrier temperature for S1 to S3; The experiment data which published on previous reports of electroluminescence measurement with different kind of heterostructure configuration to estimated thermodynamic intensive parameter.

	Normalized QE		θ_D_ (K)				T_j_ (K)			T_c_ (K)	
Samples			Shift		FWHM						
	EL	PL	EL	PL	EL	PL	V_f_	EL	PL	EL	PL
S1	1	1	825	820	830	835	560	573	578	628	616
S2	0.94	0.65	826	822	800	840	564	574	584	636	621
S3	0.85	0.42	828	823	780	860	570	578	586	691	658
Li *et al*. [35]											
Sample A	high		830	-	830	-	-	528	-	626	-
Sample B	low		827	-	820	-	-	534	-	632	-

## Conclusions

Thermodynamic intensive parameter analysis of the luminescence characteristics for GaN/InGaN MQW systems subjected to electrical injection and optical excitation was performed. The EL and PL Debye temperatures and carrier/junction temperatures were used as state variables for assessing the quantum interactions between the electrons, photons, and phonons inside structures engineered through variation of the In composition. It was found that the emission characteristics were dependent upon the InN molar fraction incorporated in the GaN/InGaN interlayers. This was the essential feature of the intensive quantities. The increase in the carrier/junction temperature with increased InN, indicated the carrier confinement to be dominated making this a potentially viable adjustable parameter for the design of heterostructures. This result, in combination with the McCumber-Sturge theory, has allowed us to develop a much better understanding of the fundamental thermophysical parameters, i.e., the EL and PL Debye temperatures, responsible for the emission quantum yield. Indeed, the EL and PL Debye temperatures, attributed to the strengths of the electron-phonon and photon-phonon interactions in the transfer of thermal energy processes, respectively, experimentally denote the enhancement or suppression in the intensity of the luminescence. Significantly, the higher luminescence efficiency was associated with higher EL Debye temperatures, lower PL Debye temperatures, and the lower carrier/junction temperatures, and vice versa. This is consistent with previous reports about the roles of the Debye and carrier/junction temperatures in luminescence characterization, these findings suggest that consideration of the intensive parameters provides an alternative approach for assessing the optical properties of a semiconductor heterostructure. Further studies are needed to develop a fully comprehensive mathematical formulation to facilitate applications and implementations of intensive-extensive analysis at the nanoscale. On the other hand, since, according to the Bose-Einstein statistics, the electron-phonon interaction coupling coefficients phenomenologically reflect the nonradiative transition rates, it also might be convenient to introduce a thermophysical marker combining the Debye temperature and the coupling coefficient to access the energy transfer phenomena in the practical application of nano-engineered technology.

## Supporting information

S1 Fig The cross-sectional TEM image for GaN/InGaN multiple quantum well heterostructures. (TIF)

S1 File The electroluminescence (EL) and photoluminescence (PL) spectra of GaN/InGaN heterostructures at an ambient temperature of 300 K. (XLSX)

S2 File The current-voltage characteristics of GaN/InGaN heterosystems with increasing InN molar fractions. (XLSX)

S3 File The emission peak energy and thermal-related FWHM from EL and PL measurement experiments. (XLSX)

S4 File The EL and PL junction temperatures obtained from the forward voltage and wavelength shift techniques and the EL and PL carrier temperatures estimated from the high-energy band tails of the InGaN luminescence spectra for GaN/InGaN blue MQB heterostrucutres. (XLSX)

S5 File The EL and PL Debye temperature extracted from the observations of the FWHM and red-shift in the emission peak as a function of tuning of the In composition in InGaN interlayers. (XLSX)
