# Supplementary figures and images for: Intensive measures of luminescence in GaN/InGaN heterostructures
Source: PLoS One. 2019 Sep 24;14(9):e0222928. doi: 10.1371/journal.pone.0222928 (PMC6759175; doi:10.1371/journal.pone.0222928)

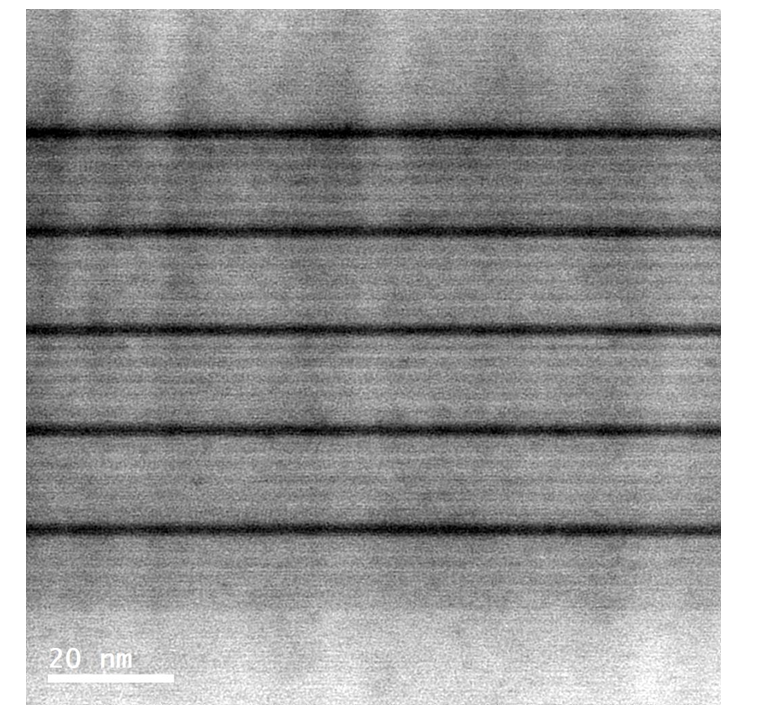

Supplement: S1 Fig — (TIF) [file pone.0222928.s001.TIF]
